# Supplementary material for: Comparison of Methicillin-Resistant Staphylococcus aureus Isolates from Cellulitis and from Osteomyelitis in a Taiwan Hospital, 2016–2018
Source: J Clin Med. 2019 Jun 7;8(6):816. doi: 10.3390/jcm8060816 (PMC6617150; doi:10.3390/jcm8060816)
Supplement: Supplementary file 1 [file jcm-08-00816-s001.pdf]

Supplementary Materials

Table S1. New *spa* types found in CL-MRSA isolates.

spa type	SCC*mec* type	MLST	*spa* repeats	PVL
txAA	IV	ST8	r11 r19 r72 r21 r17 r34 r24 r34 r22 r25	positive
txAB	IV	ST59	r04 r20 r17 r20 r17 r25 r25 r34 r34	positive
txAC	IV	ST8	r08 r21 r17 r13 r24 r34 r22 r25	negative

Abbreviations: CL: cellulitis; MRSA, methicillin-resistant *Staphylococcus aureus*; *spa*, staphylococcal protein A; SCC*mec*, staphylococcal cassette chromosome *mec*; MLST, multilocus sequence typing; ST, sequence type; PVL, Panton-Valentine leucocidin.

Table S2. Genotypes of OM-MRSA isolates in PJIs and non-PJIs.

MRSA isolates, no. (%)		MRSA isolates, no. (%)	
PJIs (n=26)		non-PJIs (n=89)	
ST239-t037-SCC*mec*III	9 (35%)	ST239-t037-SCC*mec*III	16 (18%)
		ST239-t037-SCC*mec*IV	2 (2%)
		ST239-t037-SCC*mec*V	1 (1%)
ST59-t437-SCC*mec*IV	3 (12%)	ST59-t437-SCC*mec*IV	12 (14%)
ST59-t437-SCC*mec*V	5 (19%)	ST59-t437-SCC*mec*V	7 (8%)
		ST59-t441-SCC*mec*IV	2 (2%)
		ST59-t441-SCC*mec*V	6 (7%)
		ST59-t3485-SCC*mec*IV	1 (1%)
		ST59-t8391-SCC*mec*V	1 (1%)
ST8-t008- SCC*mec*IV	3 (12%)	ST8-t008- SCC*mec*IV	15 (17%)
ST8-t008- SCC*mec*V	2 (8%)	ST8-t574- SCC*mec*IV	1 (1%)
ST8-t441- SCC*mec*IV	1 (4%)	ST8-t967- SCC*mec*IV	2 (2%)
ST5-t002- SCC*mec*II	1 (4%)	ST5-t002- SCC*mec*II	1 (1%)
		ST5-t002- SCC*mec*IV	2 (2%)
ST7-t091- SCC*mec*III	1 (4%)		
		ST9-t899- SCC*mec*IV	2 (2%)
		ST30-t019- SCC*mec*IV	2 (2%)
		ST45-t026- SCC*mec*IV	1 (1%)
		ST45-t1081- SCC*mec*IV	1 (1%)
		ST45-t1081- SCC*mec*V	5 (6%)
		ST188-t189- SCC*mec*IV	1 (1%)
		ST188-t189- SCC*mec*V	1 (1%)
		ST241-t037- SCC*mec*III	1 (1%)
		ST398-t571- SCC*mec*V	2 (2%)
ST508-t015- SCC*mec*IV	1 (4%)	ST508-t015- SCC*mec*IV	1 (1%)
		ST573-t345- SCC*mec*IV	2 (2%)
		ST573-t3525- SCC*mec*IV	1 (1%)

Abbreviations: OM, osteomyelitis; MRSA, methicillin-resistant *Staphylococcus aureus*; PJI, periprosthetic joint infections; ST, sequence type; SCC*mec*, staphylococcal cassette chromosome *mec*.

Table S3. Association between antibiotic resistance profiles and genotypes of OM-MRSA isolates from PJIs.

Antibiotic	MIC[a] (μg/mL)	MRSA isolates, no. (%)						
		ST8 (n=6)			ST59 (n=8)			ST239 (n=9)
		t008-IV (n=4)	non-t008-IV[b] (n=2)	[total] (n=6)	t437-IV (n=3)	t437-V (n=5)	[total] (n=8)	t037-III (n=9)
Ciprofloxacin	(MIC ≥ 4)	4 (100%)	0	4 (67%)	1 (33%)	1 (20%)	2 (25%)	9 (100%)
Fusidic acid	(MIC ≥ 1)	1 (25%)	0	1 (17%)	0	2 (40%)	2 (25%)	1 (11%)
Gentamicin	(MIC ≥ 16)	1 (25%)	0	1 (17%)	1 (33%)	2 (40%)	3 (38%)	9 (100%)
Rifampicin	(MIC ≥ 4)	0	0	0	0	0	0	0
TMP-SMX	(MIC ≥ 4)	0	0	0	0	0	0	9 (100%)
Vancomycin	(MIC ≥ 2)	0	0	0	0	0	0	0

[a] MIC values determined by E-test.

[b] non-t008-IV: t008-V (n=2)

Abbreviations: MIC, minimum inhibitory concentration; MRSA, methicillin-resistant *Staphylococcus aureus*; ST, sequence type; TMP-SMX, trimethoprim-sulfamethoxazole.

Table S4. Association between antibiotic resistance profiles and genotypes of OM-MRSA isolates from non-PJIs.

**Antibiotic**	**MIC[a] (μg/mL)**	**MRSA isolates, no. (%)**											
		**ST8 (n=18)**			**ST59 (n=29)**						**ST239 (n=19)**		
		**t008-IV (n=15)**	**non-t008-IV[b] (n=3)**	**[total] (n=18)**	**t437-IV (n=12)**	**t437-V (n=7)**	**t441-IV (n=2)**	**t441-V (n=6)**	**non-t437/non-t441[c] (n=2)**	**[total] (n=29)**	**t037-III (n=16)**	**non-t037-III[d] (n=3)**	**[total] (n=19)**
Ciprofloxacin	(MIC ≥ 4)	13 (87%)	3 (100%)	16 (89%)	1 (8%)	0	1 (50%)	0	0	2 (7%)	16 (100%)	3 (100%)	19 (100%)
Fusidic acid	(MIC ≥ 1)	4 (27%)	0	4 (22%)	1 (8%)	0	0	2 (33%)	1 (50%)	4 (14%)	5 (31%)	3 (100%)	8 (42%)
Gentamicin	(MIC ≥ 16)	2 (13%)	1 (33%)	3 (17%)	8 (67%)	3 (43%)	0	4 (67%)	0	15 (52%)	13 (81%)	3 (100%)	16 (84%)
Rifampicin	(MIC ≥ 4)	0	0	0	0	0	0	0	0	0	0	0	0
TMP-SMX	(MIC ≥ 4)	0	0	0	0	0	0	0	0	0	16 (100%)	3 (100%)	19 (100%)
Vancomycin	(MIC ≥ 2)	0	0	0	0	0	0	1 (17%)	0	1 (4%)	3 (19%)	1 (33%)	4 (21%)

[a] MIC values determined by E-test.

[b] non-t008-IV: t574-IV (n=1) and t967-IV (n=2)

[c] non-t437/non-t441: t3485-IV (n=1) and t8391-V (n=1)

[d] non-t037-III: t037-IV (n=2) and t037-V (n=1)

Abbreviations: MIC, minimum inhibitory concentration; MRSA, methicillin-resistant *Staphylococcus aureus*; ST, sequence type; TMP-SMX, trimethoprim-sulfamethoxazole.
